# Impact of history of esketamine treatment in the current depressive episode on response to iTBS

**DOI:** 10.1192/j.eurpsy.2025.678

**Published:** 2025-08-26

**Authors:** M. Tonkul, B. T. Baune, E. Kavakbasi

**Affiliations:** 1 Department of Psychiatry, University Hospital Münster, Münster, Germany

## Abstract

**Introduction:**

An increasing number of patients with treatment-resistant depression (TRD) are treated with a novel form of transcranial magnetic stimulation (TMS): the intermittent theta burst stimulation (iTBS). In this retrospective naturalistic study, we analyzed the outcome of iTBS treatment in patients with treatment-resistant depression.

**Objectives:**

To investigate the impact of history of esketamine treatment in the current depressive episode on response to iTBS.

**Methods:**

In this study, we included 66 hospitalized patients (57.6% female; mean age, 52.7 years) from the University Department of Psychiatry, University of Münster. Prior to iTBS treatment, 10 patients were treated with esketamine (60% female; average age, 49.8 years) and 56 were not (57% female; average age, 53.25 years). A Chi-squared test was utilized to investigate the impact of history of esketamine treatment on response to iTBS.

**Results:**

The overall response rate was 51.5%. Prior to iTBS, 15% of the patients were treated with esketamine in the current episode. In the patient group with history of esketamine treatment (ESK+), 40% of the patients responded to iTBS. In the patients without history of esketamine treatment (ESK-) in the current episode, the response rate to iTBS was 53.6 %. However, history of esketamine treatment in the current episode had no significant impact on iTBS outcome (P = 0.505; χ2 = 0,626; df = 1). The difference in baseline disease severity between the groups was not statistically significant (CGI-S 6.3 (ESK+) vs 6.1 (ESK-), P = 0.281; F = 1.184; df = 64). The total rate of treatment dropouts was 3%.

**Conclusions:**

History of esketamine treatment in the current episode was associated with worse outcome of iTBS. This finding was not statistically significant. iTBS may be an effective (40% response rate) and safe treatment for patients who did not respond to esketamine therapy.

**Disclosure of Interest:**

M. Tonkul: None Declared, B. Baune Consultant of: Received speaker/consultation fees from AstraZeneca, Lundbeck, Pfizer, Takeda, Servier, Bristol Myers Squibb, Otsuka, LivaNova, Biogen, Angelini, and Janssen-Cilag., E. Kavakbasi Consultant of: Received speaker and advisor honoraria from LivaNova and Janssen-Cilag.

